# The molecular epidemiology of human respiratory syncytial virus in a tertiary paediatric hospital between 2020 and 2024

**DOI:** 10.4102/jcmsa.v4i1.400

**Published:** 2026-10-28

**Authors:** Moepeng J. Maseko, Rageema Joseph, Stephen N.J. Korsman, Ziyaad Valley-Omar, Nei-yaun Hsiao

**Affiliations:** 1 Department of Pathology, Division of Medical Virology, Faculty of Health Sciences, University of Cape Town, Cape Town, South Africa; 2 Department of Virology, Groote Schuur Hospital, National Health Laboratory Service, Cape Town, South Africa

**Keywords:** respiratory syncytial virus, molecular epidemiology, phylogenetic analysis, genomic surveillance, paediatric respiratory infections, RSV sequences, South Africa

## Abstract

**Background:**

Respiratory syncytial virus (RSV) is a leading cause of respiratory illness in young children, contributing significantly to morbidity and mortality and placing a substantial burden on resource-limited healthcare systems. Molecular epidemiological data from sub-Saharan Africa remain limited. This study aimed to investigate the genetic diversity and evolution of RSV in a paediatric population in Cape Town, South Africa, from September 2020 to June 2024.

**Methods:**

Respiratory syncytial virus-positive respiratory samples with a cycle threshold (Ct) < 30 from patients at Red Cross War Memorial Children’s Hospital were sequenced and analysed. Phylogenetic analysis of the partial RSV genome (F and G genes) was conducted using reference sequences derived from a global sequence database. Mutational profiles were assessed for relevance to monoclonal antibody and vaccine effectiveness.

**Results:**

Of the 100 selected samples, 42 were sequenced, and 40 had sufficient coverage for analysis. Sequencing failure was associated with higher Ct values, older sample age and suboptimal storage at −20°C. Respiratory syncytial virus-A predominated (78.6%), with A.D.5.1 as the dominant lineage; RSV-B strains (16.7%) clustered within B.D.E.1 and B.D.4.1. Genotypes GA2.3.5 (RSV-A) and GB5.0.5a (RSV-B) were observed, consistent with post-pandemic global trends. G-gene analysis revealed multiple substitutions (G224E, I134K, K262E) suggestive of immune pressure. The F-gene remained more conserved; substitutions such as T122A, S211N and S389P were detected but were not linked to reduced susceptibility to monoclonals.

**Conclusion:**

This study demonstrated the co-circulation of genetically diverse RSV-A and RSV-B lineages in children between 2020 and 2024. No established resistance-associated substitutions to currently licensed RSV monoclonal antibodies were identified. Continued genomic surveillance is warranted to monitor RSV evolution.

**Contribution:**

Continued genomic surveillance is essential to strengthen molecular epidemiology and guide public health preparedness.

## Introduction

Human respiratory syncytial virus (RSV) is a major cause of severe lower respiratory tract disease in infants and young children, as well as immunocompromised adults and the elderly.^1^ The 2016 global burden of isease study estimated that RSV is responsible for 24.8 million acute respiratory tract infection episodes and 76,600 deaths each year.^2^ In low- and middle-income countries, RSV is a leading cause of mortality and morbidity in children aged less than 5 years, with approximately 60%–70% of children having been infected with RSV by the age of 1 year.^2^ In South Africa, hospital-based sentinel surveillance has reported RSV detection rates of 27% in patients under 5 years of age and 4% in adults over 18 years.^3^ Co-infection with HIV was found to affect disease outcome negatively within these populations.^3^ Hospital surveillance studies in South Africa have shown that HIV-exposed and uninfected children face a 1.4- to 2.1-fold increased risk of hospitalisation and death because of RSV compared to children who are not exposed to human immunodeficiency virus (HIV).^4^ While premature birth and existing lung or heart conditions are recognised as major risk factors for severe RSV infections, such as bronchiolitis and pneumonia, any infant can be at risk for a serious RSV lower respiratory tract infection during their first encounter with the virus.^5^ Around 91% of RSV-related hospitalisations and 99% of deaths happen in developing nations.^4^

In temperate regions, infections are more common during the winter months, while in tropical areas, they can happen throughout the year, with more variation in timing.^5^ During the Coronavirus Disease 2019 (COVID-19) pandemic, relaxation of COVID-19 restrictions led to significant out-of-season RSV outbreaks.^6^ The changes in RSV epidemiology and its resurgence could likely be attributable to diminished immunity within the community, resulting from decreased or absent exposure to the virus resulting from the imposed pandemic lockdown and non-pharmaceutical interventions.^6^ These interventions also resulted in influenza virus detections decreasing by approximately 99% from April 2020 compared to previous years, despite similar testing levels.^7^ Additionally, the genetic diversity of influenza viruses has significantly decreased, with fewer clades identified in both A(H3N2) and A(H1N1) subtypes, as well as a reduction in the B/Victoria lineage, B/Yamagata, has not been isolated since March 2020.^7^

Respiratory syncytial virus (RSV) is an enveloped, negative-sense ribonucleic acid (RNA) virus in the family *Pneumoviridae*.^8^ The RSV genome is about 15 kilobase pairs in size, encoding 11 proteins.^9^ It has two surface glycoproteins, the fusion protein (F) and the attachment protein (G). These glycoproteins play a crucial role in RSV infectivity and pathogenesis, especially the pre-F protein, while also serving as key targets for the induction of neutralising antibodies (Richman et al.).^8^ There are two antigenic groups of RSV, identified as subgroup A (RSV-A) and subgroup B (RSV-B), which were formerly categorised as subtypes. Within each subgroup, genotypes were first established based on phylogenetic clades supported by statistical analysis of the second hypervariable region of the G gene.^10^ This G gene, which encodes the attachment glycoprotein, displays the greatest genetic and antigenic variability. Recent years have seen the emergence of RSV-A (ON) and RSV-B (BA) strains, which feature a partial duplication in the distal third of the G gene.^11^ Currently, 14 genotypes have been identified in RSV-A and 24 genotypes in RSV-B.^11^ These strains have since become the predominant types circulating in many regions, but the criteria used to define these genotypes can vary, depending on the phylogenetic analysis methods employed.^12^ Valley-Omar et al. showed that in South Africa, RSV subgroup and genotype dominance shifted between seasons,^3^ and the genotype diversity was similar to that noted worldwide. This study also found common circulating RSV-A genotypes included NA1 and ON1; these were not associated with any specific observed clinical outcomes.^3^ Recently, the RSV Genotyping Consensus Consortium (RGCC), composed of experts in RSV and viral evolution, was established to standardise criteria for global RSV molecular surveillance, and their study introduced a novel classification framework for RSV below the subgroup level, designed to align with current understanding of viral diversity and evolution while ensuring practical applicability in molecular epidemiology.^10^ To classify viral lineages, they used the alias ‘D’, with A.D. representing RSV-A (historically ON1) and B.D. representing RSV-B (historically BA), followed by nested ordinal numbers, while D denotes the presence of 72-nt and 60-nt duplications within the G gene.^10^ This nomenclature system restricts nested lineages beyond three numerical levels, preventing excessive numbering, for instance, instead of extending the lineage B.D.4.1.1 indefinitely, descendant lineages are designated as B.D.E.1–B.D.E.4, where E represents 4.1.1.^10^

Respiratory syncytial virus prevention has become an increasing priority in recent years, with a new monoclonal antibody, Nirsevimab, that has been approved for the prevention of RSV disease in infants, adding to Palivizumab, which has long been the sole approved treatment for this purpose.^13^ Additionally, several vaccine candidates are in the final stages of clinical trials, with two recently approved.^13^ As these promising immunoprophylactic drugs emerge, it is crucial to develop a thorough understanding of the molecular diversity and evolutionary dynamics of RSV to enhance prevention strategies.^10^ The use of next-generation sequencing (NGS) technologies in clinical diagnostics has expanded considerably over the past few decades^14^; this advancement enables the high-throughput, massively parallel sequencing of millions of deoxyribonucleic acid (DNA) fragments.^15^ Next-generation sequencing technologies used in whole-genome sequencing can provide valuable insights into the RSV genome and its pathogenesis, enhance epidemiological surveillance and improve the understanding of RSV diversity^16^ and more effective vaccine development,^2^ as well as evaluate the effectiveness of monoclonal antibody candidates.^17^

There is a lack of comprehensive data regarding the specific strains responsible for driving the RSV epidemic in South Africa. A review of public database sequences shows disparity in the number of sequences and number of whole genomes across Africa, and there is a need for increased capacity. This study aims to investigate the genetic diversity of RSV during and after the peak of the COVID-19 pandemic, focusing on the characterisation of RSV strains infecting patients who were admitted to Red Cross War Memorial Children’s Hospital (RCWMCH) from September 2020 to June 2024, in order to gain insights into the evolution and transmission dynamics of the virus in this specific clinical setting.

## Research methods and design

### Study design and population

This was a retrospective, descriptive study of archived RSV-positive respiratory samples tested at the Groote Schuur Hospital National Health Laboratory Service (NHLS) laboratory between September 2020 and June 2024 from children (< 12 years old) who were seen at RCWMCH suffering from respiratory infections. Red Cross War Memorial Children’s Hospital is a public tertiary paediatric hospital that primarily serves low socio-economic patients in peri-urban communities and surrounding areas of Cape Town.

### Sample collection and testing

The nucleic acid from previously tested respiratory samples (tracheal aspirates, bronchoalveolar lavage, sputum and nasopharyngeal aspirates or swabs) was archived, as is standard laboratory practice. Data extraction from the Laboratory Information System (LIS), as well as a manual search of worksheets, was conducted in order to retrieve the sample episode number, cycle threshold (Ct)^18^ and associated demographic information. A list of 718 RSV-positive respiratory samples was identified from 5592 respiratory samples reviewed, and eligible samples were selected using purposive sampling with a target of approximately 20 samples per study year, with a target of 100 RSV-positive samples.

Samples included in the study were previously tested for RSV using the Seegene Allplex RV Essential and Anyplex RV16 real-time polymerase chain reaction (PCR) assays (Seegene, Seoul, South Korea). Only samples with a Ct value below 30 were considered for inclusion in the analysis. Where an eligible specimen was unavailable because no residual eluate remained or the remaining volume was insufficient, the next eligible specimen on the chronological list for the study year was selected until the target sample number was achieved.

As this was an exploratory descriptive genomic study, no formal sample size or statistical power calculation was performed. A pragmatic sample of 100 archived RSV-positive samples (approximately 20 per study year) was selected based on specimen availability, temporal representation across the study period and available sequencing resources.

Selected RSV sample eluates had been stored at −20°C and/or −70°C storage. Samples were extracted using the BioMérieux Nuclisens Easymag extraction kit (Marcy-L’Etoile, France) in accordance with the manufacturer’s instructions, with an extracted sample and elution volume of 500 µL and 50 µL, respectively.

### PCR and reverse transcription

Complementary DNA (cDNA) strands were synthesised from RNA templates and target DNA subsequently amplified using the SuperScript IV One-step Reverse Transcription Kit (ThermoFisher Scientific, Carlsbad, CA). Each RSV sample required four independent sets of primers/PCR reactions, one primer set for each fragment of the genome.

The primer sets used are from Bose et al. (2015)^19^ and Schobel SA et al. (2016)^20^:

Primer set 1: forward 5-GGCAAATAAGAATTTGATAAGTAC-3 and reverse 5-TTTGATTGMAAAAWCGTGTAGCTG-3, Primer set 2: forward 5-GCCACARAGTCAATTYATAGTAG-3 and reverse 5-TGTRACTGGTGTGYTTYTGG-3, Primer set 3: forward 5-TGATGCATCAATATCTCAAGTCA-3 and reverse 5-GRCCTATDCCTGCATACTC-3 and Primer set 4: forward 5-TGGACCATWGAAGCYATATCA-3) and reverse 5-AGTGTCAAAAACTAATRTCTCGT-3.

Two master mixes (MM) were prepared for the one-step reverse transcriptase (RT)-PCR protocol. Master Mix 1 (MM1) consisted of 1.25 µL each of forward and reverse primers (20 µM) and 2.5 µL of nuclease-free water, to which 5 µL of RSV sample eluate was added. Master Mix 2 (MM2) contained 12.5 µL of SuperScript IV One-Step RT-PCR 2X buffer, 0.5 µL of SuperScript IV RT mix and 1.5 µL of nuclease-free water. Thermal cycling conditions and reaction setup were adapted from the protocol described by Dong et al.^21^ Once complete, PCR products were run on a 1% agarose gel electrophoresis to confirm successful fragment amplification. PCR products were quantified and pooled in equimolar concentrations using the Qubit Fluorometer with the Qubit 1x dsDNA HS assay kit (ThermoFisher Scientific, Carlsbad, CA).

### Library preparation and respiratory syncytial virus sequencing

Library preparation was performed using the Illumina COVIDSeq protocol (Illumina, Document #1000000128490 v03), adapted for RSV whole-genome amplicons. The primary modification to the original protocol was the use of quarter reaction volumes for all library preparation steps, rather than the full reaction volumes specified. Following amplification, pooled RSV genomic amplicons were enzymatically fragmented and end-repaired. Unique dual indexing adapters were ligated to each fragment using the Illumina DNA Prep chemistry, and a second round of PCR amplification was carried out to incorporate sample-specific indexes. The resulting indexed libraries were pooled and underwent bead-based size selection for purification. Library quality was assessed via agarose gel electrophoresis, and concentration was quantified using the Qubit dsDNA High Sensitivity assay on a Qubit fluorometer (ThermoFisher Scientific, Carlsbad, California, United States). The purified library pool was normalised to 4 nM concentration and denatured with 0.2 N sodium acetate. The 4 pM sample library was spiked with 1% PhiX Control v.3 adaptor-ligated library. The pooled library was sequenced on a 500-cycle v.2 MiSeq Reagent Kit on the Illumina MiSeq instrument (Illumina, Inc., San Diego, California, United States) using sequencing by synthesis (SBS) chemistry.

### Nucleotide sequence analysis

For genome assembly, raw paired-end nucleotide sequences were analysed, edited and aligned using the Genome Detective online bioinformatic software (Emweb, Herent, Belgium; https://www.genomedetective.com/app/typingtool/virus/). Raw sequence reads generated by the Illumina MiSeq platform were uploaded to the Genome Detective website in .fastq.gz format. The pipeline removed low quality as well as human DNA reads prior to assembly. The remaining reads were assembled to appropriate RSV reference sequences. The nucleotide sequence typing of samples was done using the Nextstrain tool (https://clades.nextstrain.org/), specifically the Nextclade typing tool for clade determination and genome coverage generation. Nextstrain integrates data curation, analysis and visualisation to track viral evolution. It uses Python scripts to maintain a database of genomic sequences and metadata from public repositories such as National Center for Biotechnology Information (NCBI), Global Initiative on Sharing All Influenza Data (GISAID) and Virus Pathogen Resource (ViPR), along with other sources^22^ Its analytical tools perform phylodynamic analyses, which include sequence subsampling, alignment, phylogenetic inference, temporal dating of ancestral nodes and geographic reconstruction of transmission events.^22^ Global and South African RSV sequences available on the GISAID database between January 2020 and December 2024 were downloaded, and RSV-A and RSV-B control datasets were generated. Sequences were aligned using BioEdit Software version 7.1.3. Maximum-likelihood phylogenetic trees were constructed using MEGA version 11.0.13 software with bootstrap replicates of 1000.

### Ethical considerations

The study protocol was approved by the Health Sciences Human Research Ethics Committee (HREC) of the University of Cape Town (approval number: HREC 547/2023). In addition, permission to access and retrieve samples from the NHLS was granted by the Academic Affairs Research Management System (AARMS) approval number PR2556697. The study utilised routinely tested and resulted diagnostic samples, with no additional sample collection. Informed consent was waived by the ethics committee, as the study involved retrospective analysis of stored, de-identified samples. All data were anonymised and managed in accordance with institutional data protection policies. All procedures adhered to the relevant institutional guidelines and standard operating procedures.

## Results

PCR was performed on 100 selected samples, with 20 samples per year included, provided they met the study’s inclusion criteria. Forty-two successfully amplified respiratory samples from children with PCR-confirmed RSV acute lower respiratory tract infection were included during the study period from September 2020 to June 2024. The median age of the children’s samples included in the study was 2 months, with an interquartile range of 5 months. Fifty-two per cent of those included were male, and 48% received care in the Intensive Care Unit (ICU). Only 6 (14%) of the children had HIV exposure, with only 1 (2%) child having a positive result.

Samples regarded as successfully sequenced had a genome coverage of more than 70% (Figure 1). Of the 42 samples sequenced, 40 were successful. The distribution of successfully sequenced samples across study years is shown in Online Appendix 1, Figure 1-OA1.The highest number of sequences was obtained from 2024, with 16 successfully sequenced samples, followed by 10 from 2020, 6 from 2021 and 5 from 2023. In 2022, five samples were processed, but only three yielded successful sequencing results. In total, 40 samples were successfully sequenced and subtyped across the study period. Most successfully sequenced samples had Ct values below 20, with only 14 out of 40 exhibiting Ct values above this threshold. Notably, most of the successfully sequenced samples with Ct values above 20 were from 2024, whereas older samples required lower Ct values for successful sequencing.

**FIGURE 1:**
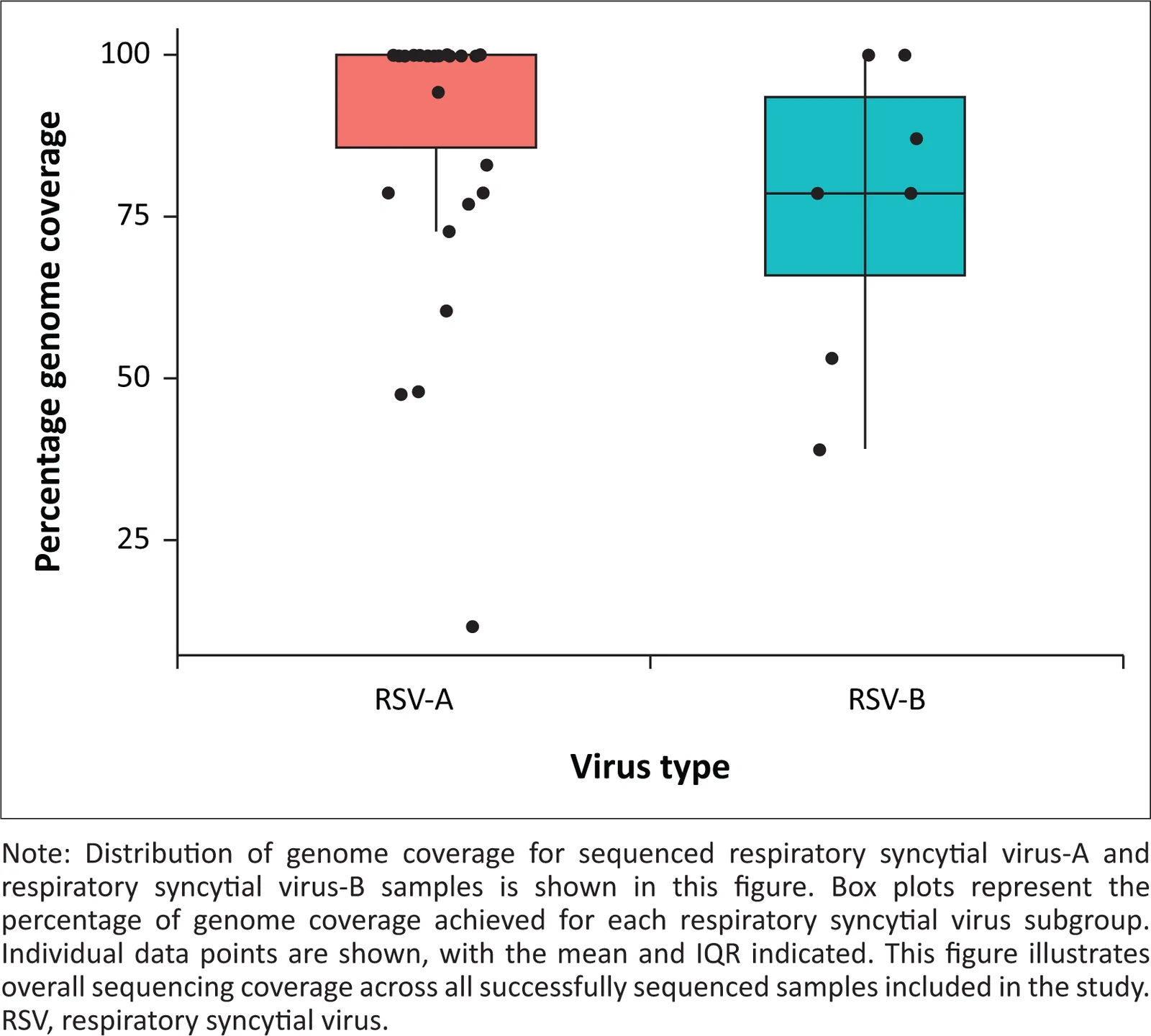
Genome coverage of sequenced respiratory syncytial virus-A and respiratory syncytial virus-B samples.

The temporal distribution of sequenced samples in relation to the COVID-19 waves is illustrated in Figure 2. Of the 40 samples that were successfully typed and sequenced, seven were collected shortly after the first COVID-19 wave, which peaked between epidemiological weeks 24 and 28 in 2020. Four samples were obtained during the second wave, which peaked starting in week 47 of 2020, extending into the first week of 2021.^23^ An additional three samples were collected immediately following the second wave in 2021. Two samples from 2021 were obtained shortly after the fourth wave, which peaked in December 2021.^18^ One sample was collected during the peak of the fifth wave in May 2022, and two additional samples were collected in the post-wave period that followed.^18^ The remaining 21 samples were collected during 2023 and 2024, after the conclusion of all major COVID-19 waves. These sampling periods overlap with and extend beyond typical RSV seasonality observed in South Africa, where RSV epidemics generally occur just before or during the winter months. The timing of sample collection thus reflects both pandemic-related shifts in RSV circulation and its expected seasonal trends.

**FIGURE 2:**
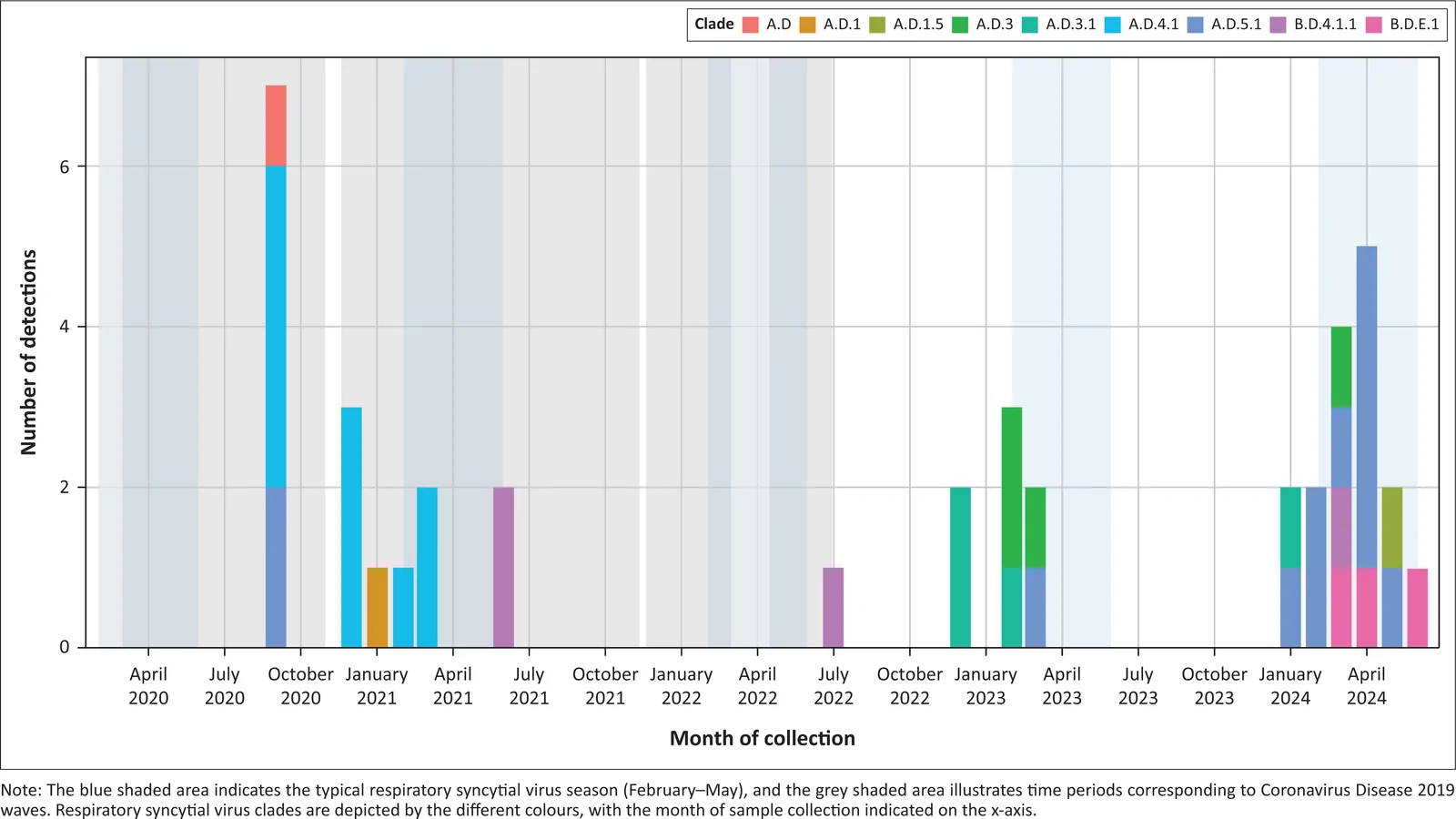
Respiratory syncytial virus clade circulation in South Africa in relation to seasonal respiratory syncytial virus activity (February–May) with Coronavirus Disease 2019 waves.

### Genome sequencing and assembly

The box and whisker plot (Figure 1) summarises the percentage genome coverage for sequenced RSV-A and RSV-B samples. For RSV-A, the median coverage is 99.8% (interquartile range [IQR] 85.7% – 99.8%). Three RSV-A sequences had very low coverage (11%, 47% and 48%). For RSV-B, the median coverage is 78.5% (IQR 65.8% – 93.4%). Among the 42 samples sequenced, 83% (35/42) yielded RSV genomes with more than 70% coverage. Specifically, 27 samples had ≥ 99% genome coverage, 2 had > 82% coverage, 5 had > 76% coverage and 1 had > 72% coverage. Subgroup analysis of the total samples sequenced revealed that 33 out of 42 sequenced samples were classified as RSV-A, while 7 were identified as RSV-B. Two samples failed sequencing and could not be subtyped. Among the 42 (40 successfully subtyped) sequenced samples, 38 (32 RSV-A and 6 RSV-B) exhibited complete G gene coverage, while 33 (27 RSV-A and 6 RSV-B) had full F gene coverage.

### Clade assignment and genetic diversity

The RSV lineages identified in this study belonged to nine distinct clades. Among RSV-A samples, the majority clustered within A.D.5.1 totalling 12 (36%) samples, and A.D.4.1 with 10(30%), while A.D.3 and A.D.3.1 were represented to a lesser extent with 4 (12%) samples each. Additionally, A.D.1.5, A.D.1 and A.D. each accounted for a single sample (3%). In contrast, only two clades were observed for RSV-B: B.D.4.1.1 with four (57%) samples and B.D.E.1 with three (43%) samples (Online Appendix 1 Table 1-OA1).These lineages align with the previously recognised ON1 genotype, and all 33 RSV-A samples clustered within the GA2.3.5 G-clade based on G-gene analysis. Similarly, all seven RSV-B samples were classified within the GB5.0.5a G-clade, corresponding to the previously recognised BA9 genotype.

The annual distribution of RSV clades detected from 2020 to 2024 is illustrated in Figure 2. Clade A.D.5.1 was the most frequently identified, particularly in 2020 and 2024. Other clades, including A.D.3.1, A.D.4.1 and B.D.E.1, were detected intermittently across different years. The data show a shifting pattern of RSV clade circulation over time, with certain clades emerging or declining in prevalence. Notably, multiple clades co-circulated in some years, reflecting the genetic diversity of RSV strains detected in this study. In 2024, RSV-A clades A.D.3.1 and A.D.5.1 and RSV-B clades B.D.E.1 and B.D.4.1.1 were circulating simultaneously.

### Maximum likelihood phylogenetic tree of respiratory syncytial virusA G- and F-gene

Of the 33 RSV-A genomes analysed, 32 had sufficient G-gene coverage and were included in the phylogenetic analysis (Figure 3), marked with red dots. The tree demonstrates the co-circulation of diverse RSV-A lineages over time, with notable clustering within A.D.5.1, A.D.4.1 and A.D.3, with multiple sequences forming tight clusters supported by high bootstrap values. The clustering reflects both clonal expansion and localised diversification during the study period. Within A.D.5.1, seen mostly in 2024, several study samples occupy terminal branches, suggesting its continued expansion and dominance in recent RSV seasons and possible ongoing transmission. A.D.4.1 and A.D.3 demonstrate sub-clustering, including different time points, from 2020 to 2024, demonstrating their persistence across multiple seasons. Study sequences are interspersed among globally reported RSV-A strains from South Africa, the UK, Switzerland and China, reflecting ongoing international transmission and shared evolutionary origins. Older sequences (pre-2020) cluster closer to the root, while recent strains (2023–2024) appear in more divergent branches, indicating continued viral evolution. High bootstrap support indicates strong reliability of the observed relationships between sequences while also reinforcing the robustness of phylogenetic clustering of key branches and supporting the accuracy of lineage assignments, confirming the genetic relationships among RSV-A sequences. These findings highlight the dynamic circulation of RSV-A lineages over time, with newer lineages like A.D.5.1 expanding in 2024 and earlier lineages (A.D.3 and A.D.4.1) persisting over multiple seasons.

**FIGURE 3:**
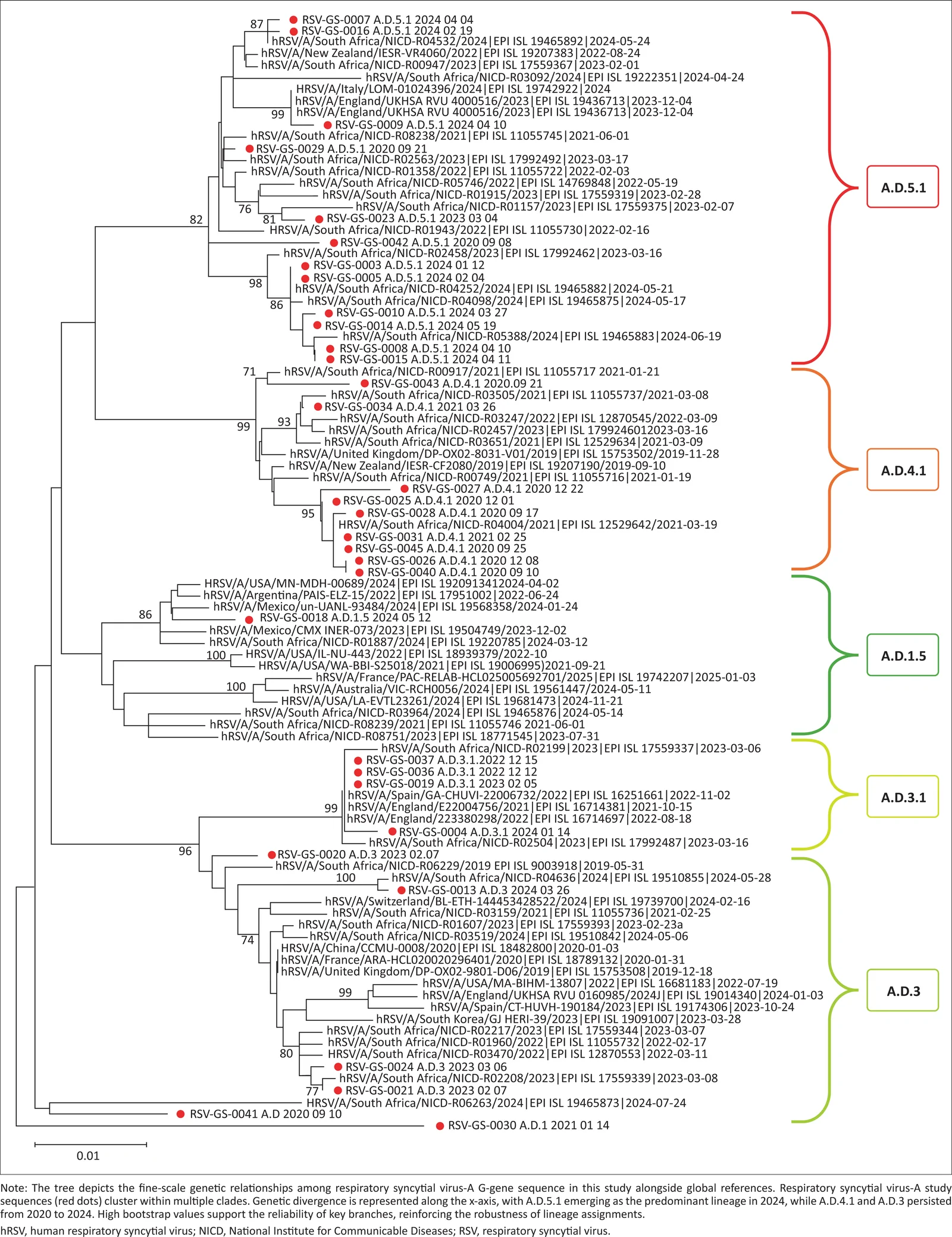
Maximum likelihood phylogenetic tree of respiratory syncytial virus-A G-gene sequence between 2020 and 2024.

A maximum likelihood phylogenetic tree of the RSV-A F-gene (Figure 4), including both study samples (marked with blue dots) and reference sequences from local and global databases, was constructed using 27 study samples (five samples were excluded because of inadequate F-gene coverage). Study sequences are distributed across multiple clades, with a significant number clustering within the A.D.5.1 lineage.

**FIGURE 4:**
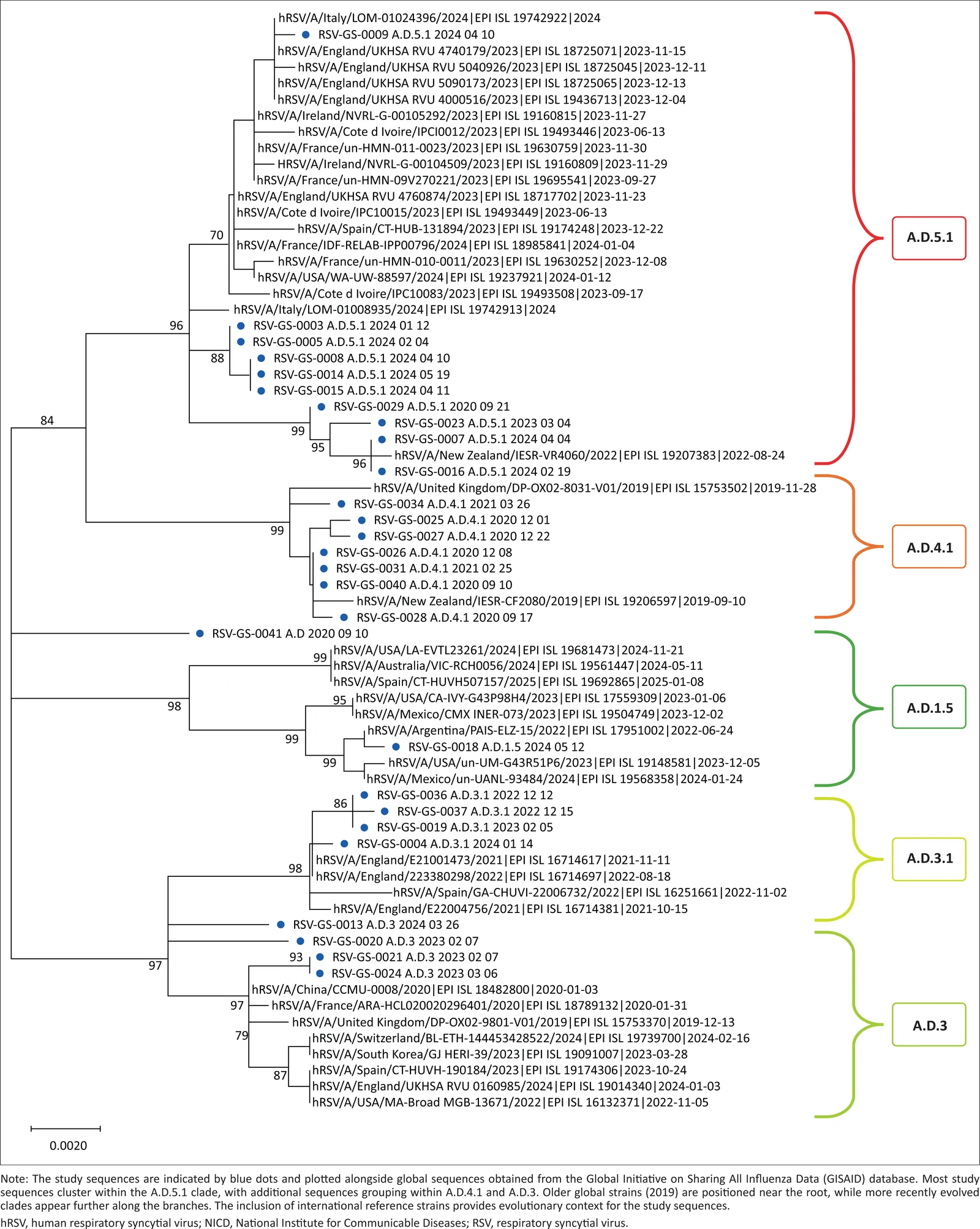
Maximum likelihood phylogenetic tree illustrates the evolutionary relationships among respiratory syncytial virus-A F-gene sequences collected from 2020 to 2024, with genetic divergence represented along the x-axis.

The A.D.5.1 clade appears to be one of the dominant lineages, with multiple study sequences from 2024 positioned alongside global RSV-A strains from Italy, the UK and Ireland. Some sequences also fall within A.D.4.1 and A.D.3, showing continued circulation of these clades. High bootstrap values (e.g. 99, 98) support the reliability of many branch groupings, indicating strong phylogenetic confidence.

Regarding global circulation, study sequences are interspersed among globally reported RSV-A strains, including those from the UK, Italy, Spain, France, New Zealand, Australia and China.

The presence of multiple international strains within close evolutionary proximity suggests continued RSV-A circulation and potential global transmission links. The A.D.5.1 lineage has multiple study samples from 2024, reinforcing its persistence and expansion. Some study sequences from earlier years (e.g. 2020, 2022 and 2023) are positioned within A.D.3 and A.D.4.1, indicating these clades were circulating before A.D.5.1 became dominant. The clustering of study sequences with global strains suggests shared evolutionary origins rather than entirely distinct local evolution. With the exception of one sample, all study sequences clustered with globally circulating clades from the corresponding time period, which may represent incomplete sequence data, as the clade could not be assigned.

### Maximum likelihood phylogenetic tree of respiratory syncytial virus-B G- and F-gene

The RSV-B G-gene phylogenetic tree reveals that study sequences cluster within the B.D.4.1.1 and B.D.E.1 clades, which are the dominant RSV-B lineages circulating during the study period (Figure 5). Study sequences closely group with global RSV-B strains from Europe (Germany, Spain, Italy), North America (United States, Canada) and Asia (China, Qatar), suggesting shared evolutionary origins and potential transmission links. B.D.E.1 sequences show greater genetic divergence, indicating ongoing evolution and diversification within this lineage. The presence of high bootstrap values (e.g. 99, 97) supports the reliability of the clade assignments. Study samples from 2024 (B.D.E.1) and earlier samples from 2021 to 2022 (B.D.4.1.1) demonstrate the persistence and expansion of these lineages over multiple seasons.

**FIGURE 5:**
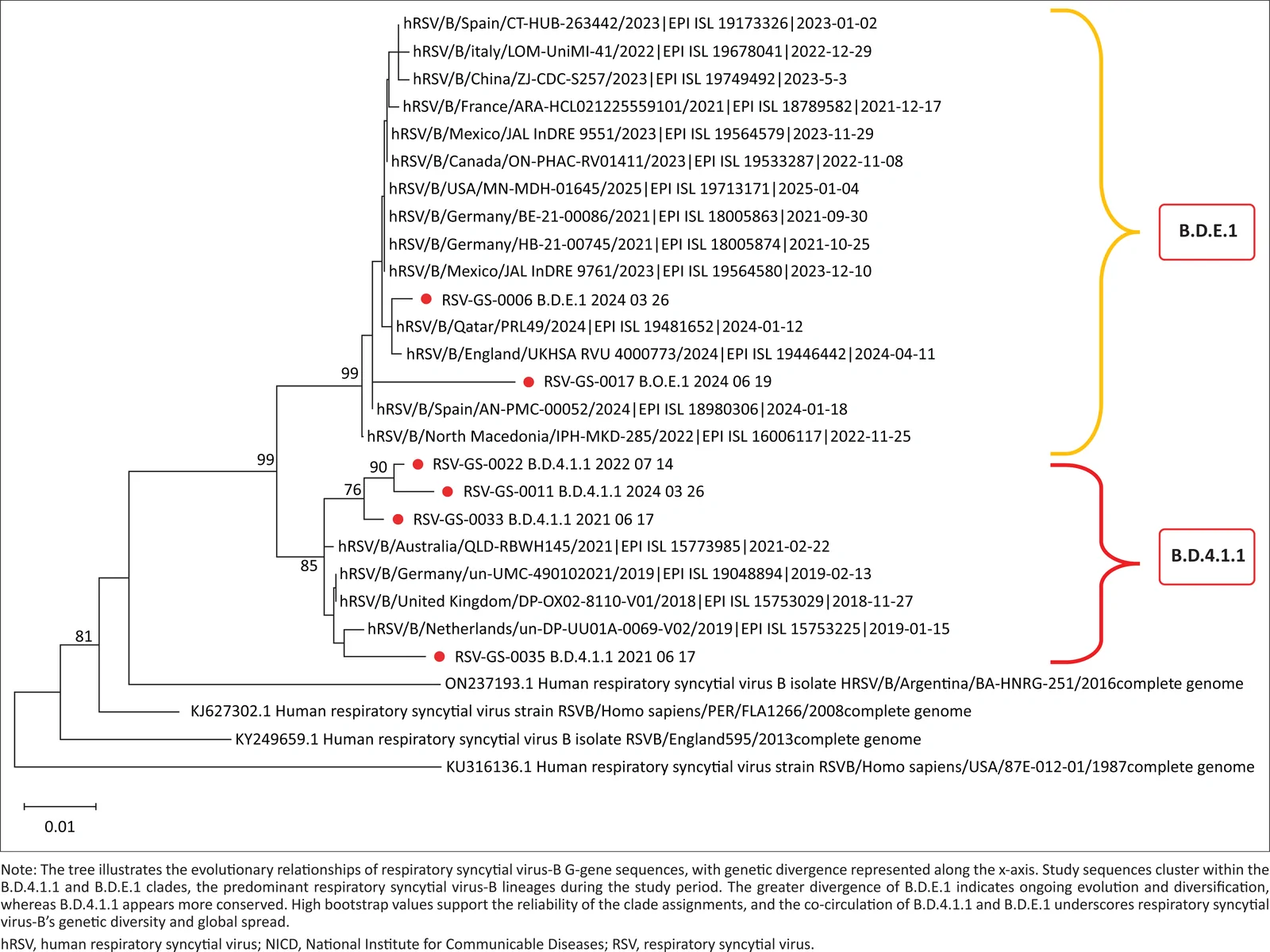
Maximum likelihood phylogenetic tree of respiratory syncytial virus-B G-gene sequences (2020–2024).

The RSV-B F-gene phylogenetic tree shows that study sequences are also distributed across B.D.4.1.1 and B.D.E.1, consistent with G gene clustering (Figure 6). The presence of study sequences within well-supported clades (bootstrap values ≥ 97) confirms their genetic relationship to contemporary RSV-B strains. Minimal divergence is observed in the F-gene compared to the G-gene, suggesting that the F protein remains relatively conserved, likely because of functional constraints. Study sequences cluster with global RSV-B strains from Europe, North America and Asia, reinforcing the ongoing circulation of these lineages internationally. Co-circulation of B.D.4.1.1 and B.D.E.1 in 2024 aligns with patterns observed in the G-gene tree, highlighting simultaneous circulation of multiple RSV-B variants.

**FIGURE 6:**
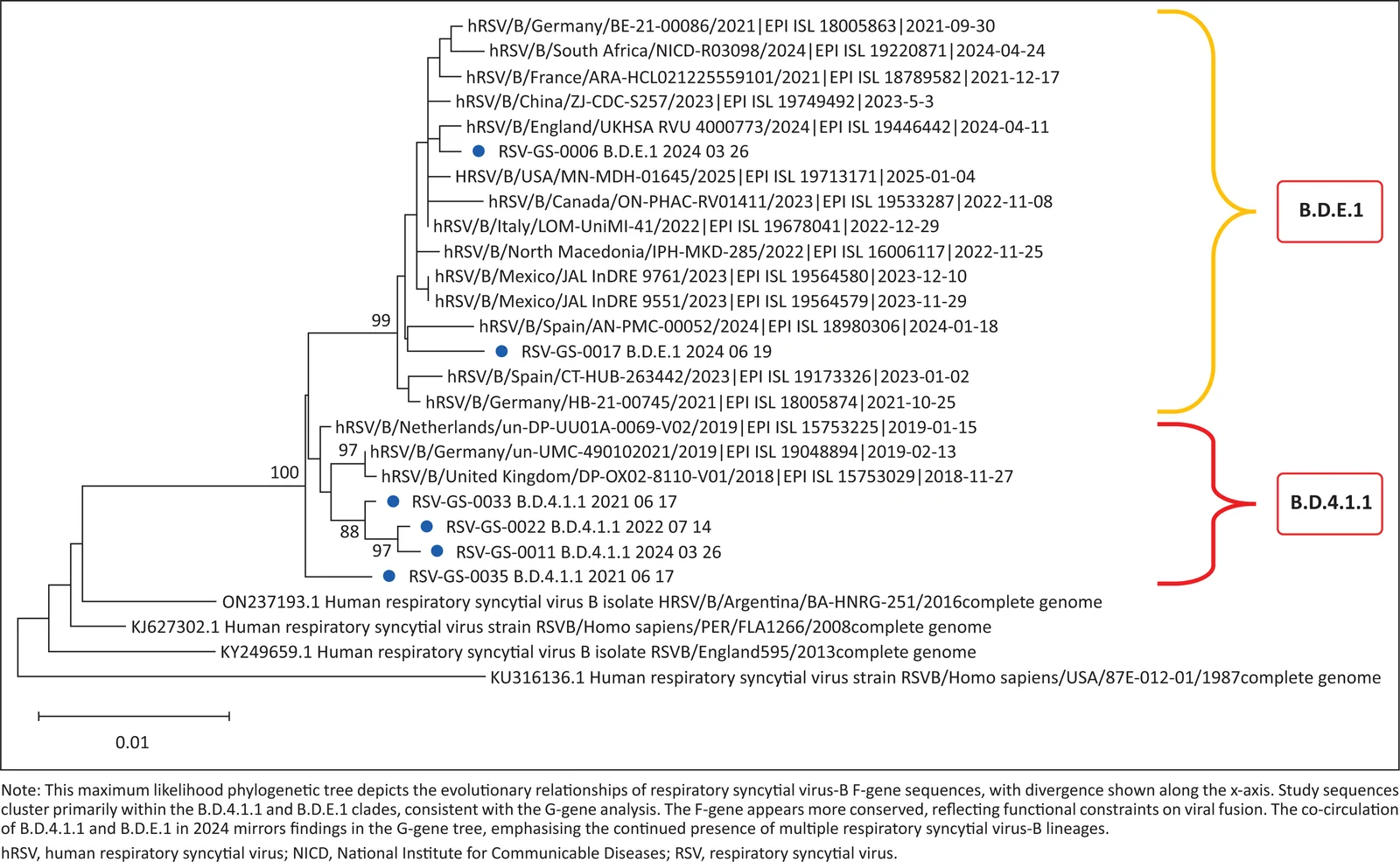
Phylogenetic tree of respiratory syncytial virus-B F-gene sequences (2020–2024).

Both phylogenetic analyses confirm co-circulation of RSV-B clades B.D.4.1.1 and B.D.E.1, with B.D.E.1 showing higher genetic divergence in the G-gene. Study sequences align closely with globally reported strains, suggesting widespread distribution and continued transmission of these clades.

The F-gene remains more conserved than the G-gene, consistent with its structural and functional constraints.

### G-gene amino acid substitutions

The RSV-A G-gene exhibits a high frequency of P71L, I134K, S234I and G224E, with clade-specific variations in A.D.1, A.D.1.5, A.D.3.1 and A.D.4.1. The RSV-B G-gene shows universal presence of A74V, with additional mutations occurring at lower frequencies. These findings highlight genetic divergence and potential antigenic variability, emphasising the need for continued surveillance of RSV-A and RSV-B evolution. A detailed table of mutations (Online Appendix 1 Table 2-OA1 and Table 3-OA1) is provided in the supplementary data section.

### F-gene amino acid substitutions

Several notable amino acid substitutions in the RSV F protein, including T122A, K123Q, S211N, I384T and S389P, have been identified in circulating RSV strains in this study (Table 1), some of which are linked to immune evasion and potential effects on neutralisation by monoclonal antibodies (mAbs) and vaccines.

**TABLE 1:** Amino acid substitutions identified within the F-gene of respiratory syncytial virus-A and respiratory syncytial virus-B.

Substitution	Total		A.D.	A.D.1	A.D.1.5	A.D.3	A.D.3.1	A.D.4.1	A.D.5.1	B.D.4.1.1	B.D.E.1
	*n*	%									
**RSV-A**											
T12I	8	26	0	0	0	4	4	0	0	-	-
T122A	20	65	0	0	0	0	0	10	10	-	-
K123Q	9	29	0	0	0	0	0	9	0	-	-
I384T	9	29	0	0	0	0	0	9	0	-	-
**RSV-B**											
T211I	1	17	-	-	-	-	-	-	-	1	0
S190N	2	33	-	-	-	-	-	-	-	0	2
S211N	3	50	-	-	-	-	-	-	-	1	2
S389P	4	67	-	-	-	-	-	-	-	2	2

Note: Total samples with amino acid substitutions within different lineages.

RSV, respiratory syncytial virus.

#### Respiratory syncytial virus-A F-gene mutations

T122A (65%) was restricted to A.D.4.1 and A.D.5.1, while K123Q and I384T (29%) were found only in A.D.4.1. T12I (26%) was unique to A.D.3 and A.D.3.1.

#### Respiratory syncytial virus-B F-gene mutations

S389P (67%) was present in all B.D.4.1.1 and B.D.E.1 sequences, while S211N (50%), S190N (33%) and T211I (17%) were also observed.

F-gene analysis of RSV-A revealed four substitutions compared to the reference sequences. T122A was present in 65% of samples but only in two clades A.D.4.1 and A.D.5.1. K123Q and I384T were present in 29% of samples but only in clade A.D.4.1. T12I was present in 26% of samples but only in clades A.D.3 and A.D.3.1.

Respiratory syncytial virus-B F-gene analysis showed four substitutions. S389P was present in 67% of samples and in clades B.D.4.1.1 and B.D.E.1. S211N, S190N and T211I were present in 50%, 33% and 17%, respectively. Among the substitutions reported to affect monoclonal antibody binding activity, only one, S276N, was identified. This substitution, which is known to impact the activity of palivizumab and nirsevimab, was detected in four (13%) of the RSV-A samples. The other substitutions, N201K, R209Q and R209K, which have been reported to have some impact, were not detected.^17^

## Discussion

This study demonstrated the co-circulation of multiple RSV strains, with a predominance of RSV-A (78.6%) over RSV-B (16.7%) between 2020 and 2024. These findings are consistent with previous reports highlighting the cyclical dominance of one subtype over another. However, based on a limited sample size, the results align with established patterns of RSV circulation. Limited genomic surveillance prior to the COVID-19 pandemic may have led to an under-representation of certain RSV lineages.^10^ Some lineages, including A.D.3.1, A.D.5.2 and A.D.5.3 in RSV-A, as well as B.D.E.1 and B.D.E.3 in RSV-B, appear to have emerged exclusively in the post-pandemic period.^10^ However, many of their defining amino acid residues were already present in ancestral pre-pandemic strains.^10^ In this study, sequence analysis identified the presence of A.D.3.1 and B.D.E.1. These observations are consistent with what has been seen in other studies in South Africa, where RSV-A dominates while co-circulating with RSV-B.^16,24^

Recent global studies indicate that GA2.3.5 and GB5.0.5a genotypes have been the predominant genotypes circulating both before and after the COVID-19 pandemic.^16^ This pandemic, as well as the implementing of non-pharmaceutical interventions (NPIs), significantly impacted RSV transmission within communities; RSV pre-pandemic epidemics followed a seasonal pattern but were disrupted during the pandemic because of NPIs, leading to minimal detection of RSV.^25^ The surge of cases post pandemic was attributed to strains circulating prior to the pandemic, and similar patterns have been seen worldwide.^16^ The observed genotype distributions in this study aligned with both local and international RSV circulating patterns (Figure 3, Figure 4, Figure 5, and Figure 6). This finding may suggest that RSV continued to circulate even during periods of low or undetected incidence, representing asymptomatic transmission or an unsampled population.^25^ Phylogenetic analyses of the F- and G-genes revealed that co-circulating RSV strains belonged to known lineages, with no novel variants detected (Figure 3 to Figure 6). The dominant genotypes seen in this study were GA2.3.5 for RSV-A and GB5.0.5a for RSV-B, which correspond to the previously recognised ON1 and BA9 genotypes, respectively. ON1 was first recognised in Ontario, Canada, in 2010 and since then has been reported in many countries worldwide,^26^ eventually becoming the dominant genotype in South Africa, with prevalence increasing from 5.6% in 2012 to 81.5% in 2015.^3^ These findings are consistent with recent South African genomic studies conducted during and after the COVID-19 pandemic, which similarly identified GA2.3.2/GA2.3.5 and GB5.0.5a as the predominant RSV-A and RSV-B genotypes, respectively, circulating in hospitalised children in the Free State.^16^ The lengthening of the G protein in ON1 and BA9 genotypes, resulting from 72- and 60-nucleotide duplications, respectively, is believed to have contributed to their rapid global dissemination, and it is hypothesised that these structural modifications confer fitness and evolutionary advantages, facilitating their dominance over other genotypes.^26^

Respiratory syncytial virus evolution is primarily driven by immune pressure and the error-prone nature of its RNA-dependent RNA polymerase, which lacks proofreading ability.^27^ Consistent with previous studies, we identified sequence duplications in the second variable region of the G protein. This region contains key antigenic epitopes and plays a role in the emergence of new variants, particularly under immune pressure following natural infections.^26^ Furthermore, duplications in this region extend the length of the G protein and alter its structure, potentially enhancing viral attachment and immune evasion.^26^ The extracellular domain of the G protein contains two highly glycosylated regions (aa66-160 and aa192-319) that are known to be highly variable.^28,29^ These regions play a crucial role in RSV pathogenicity and immune evasion. The substitutions in our study samples occurred in this domain. Analysis of amino acid substitutions in the G-gene of RSV-A identified 14 substitutions within GA2.3.5 samples. Three of these substitutions – I134K (100%), S243I (100%) and K262E (94%) – are likely reversion mutations, as previously described in RSV antigenic variation. These mutations may have arisen to alter antigenicity, given that older ON1 strains carried the reverse substitutions (K134I, I243S and E262K).^28,29^ This may contribute to the short-lived immunity and the frequent occurrence of recurrent infections. Site E224 has been identified as highly susceptible to mutations because of immune pressure, as observed in an Iranian cohort from 2018–2019 and an Italian cohort, where E224G and E224V substitutions were reported.^30,31^ In the present study, the G224E substitution was detected in 97% of samples, suggesting it is likely a reversion mutation driven by immune pressure.

In the RSV-B amino acid G-gene analysis, A47V was detected in 100% of the cohort of samples in this study and was also detected in all samples in the cohort tested at Johns Hopkins Health System in the United States.^29^ Similar to the United States cohort, this study also detected the S100G (33%) substitution, which has become prominent within the RSV-B genome, indicating a potential fitness advantage.^29^

While the F-gene is highly conserved, four substitutions were identified in both RSV-A and RSV-B. In RSV-A, the T122A substitution was detected in 65% of samples in this cohort. A previous study in South Africa reported a reversion substitution, A122T, between 2019 and 2020,^24^ indicating that this site undergoes changes over time. However, its impact on F-protein function remains unknown.

Among the amino acid substitutions identified in the RSV-B F-gene, S211N and S389P are located within antigenic sites targeted by nirsevimab and palivizumab, respectively.^17,24^ There is no effect of these substitutions on monoclonal antibody efficacy, and currently they remain effective. These substitutions have become common in South African cohorts.^24^

Respiratory syncytial virus-A mutations in the p27 region (T122A, K123Q) have been linked to immune escape, but vaccine-induced immunity is broadly protective, reducing the risk of antigenic drift.^17,24^

Respiratory syncytial virus-B mutations (S211N, S389P) have become dominant globally, yet no vaccine breakthrough cases have been reported because of these substitutions.^17,24^

The genetic diversity of RSV-A and RSV-B F-gene sequences highlights ongoing viral evolution, but current vaccines and antibody therapies remain largely effective. The presence of potentially immune-evasive mutations, particularly in antigenic sites and the p27 region, underscores the need for continued genomic surveillance to assess their long-term impact on RSV immunity and treatment strategies. No known mutations associated with reduced monoclonal antibody binding were detected in this study; however, the small sample size limits the ability to draw definitive conclusions.

### Factors associated with success or failure to sequence as observed in this study

In this study, several factors were observed to influence the success or failure of amplification and sequencing. Of the 100 selected samples that met inclusion criteria, only 42 were successfully amplified, while the remainder failed PCR amplification. Sequencing success appeared to be strongly influenced by factors affecting RNA integrity and quantity. Higher Ct values, reflecting lower viral load, were associated with failure. In addition, conditions such as sample age and suboptimal storage contributed to RNA degradation, reducing the likelihood of successful amplification. Notably, most of the successful 2020 samples were stored at −70°C and had Ct values in the high teens, whereas samples with lower Ct values but stored at −20°C were more prone to amplification failure, particularly if they were older. Furthermore, the number of freeze–thaw cycles impacted amplification success. Samples that had undergone multiple freeze–thaw cycles or had been re-tested multiple times were more likely to fail PCR amplification, suggesting that repeated handling and temperature fluctuations contributed to RNA degradation. These findings emphasise the importance of optimal sample storage, minimal freeze–thaw cycles and prioritising lower Ct value samples for successful sequencing. These observations highlight the inherent instability of RNA and the critical role of appropriate storage conditions, particularly temperature maintenance, in preserving sample integrity for sequencing.

### Limitations of the study

This study has several limitations. The sample size was relatively small (42 samples), with only 35 having sufficient genome coverage for phylogenetic and mutational analyses. Additionally, the study was limited to samples from RCWMCH in Cape Town, restricting geographic diversity and potentially limiting the generalisability of findings for a broader molecular epidemiological analysis. In addition, the study was not powered for inferential comparisons between RSV subgroups, lineages or clinical characteristics. Although several RSV lineages were detected, the small, single-centre sample cannot be assumed to represent the full diversity or relative prevalence of RSV lineages circulating in the broader population. The use of RSV-positive eluates instead of primary samples may have impacted sequencing success, while RNA degradation and suboptimal storage conditions after initial diagnostic testing likely contributed to PCR amplification failures.

Furthermore, the narrow sample pool and variability in sample stability may have influenced sequencing efficiency, particularly for samples with higher Ct values (higher Ct values are used as a proxy to represent lower viral loads), reducing the probability of successful genome amplification. These factors highlight the need for larger, more geographically diverse sample sets, improved sample preservation and optimised sequencing approaches in future studies.

## Conclusion

This single-centre study demonstrated the co-circulation of genetically diverse RSV-A and RSV-B lineages in children between 2020 and 2024. The study sequences clustered with contemporary South African and international lineages, while the G gene showed greater variability than the more conserved F gene. Although amino acid substitutions occurred at sites of potential antigenic relevance, genomic findings alone cannot determine vaccine or monoclonal-antibody effectiveness. No established resistance-associated substitutions were identified in this limited sample. Larger, systemically sampled, multicentre studies are required to determine the epidemiological and clinical significance of these variants.
